# Effect of β-cyclodextrin deodorization on the volatile chemicals and functional properties of three types of gelatins

**DOI:** 10.3389/fnut.2022.1059403

**Published:** 2022-11-07

**Authors:** Lili Yang, Ye Zi, Cuiping Shi, Jiahui Chen, Jiamin Xu, Xichang Wang, Jian Zhong

**Affiliations:** ^1^Shanghai Key Laboratory of Pediatric Gastroenterology and Nutrition, Xinhua Hospital, Shanghai Institute for Pediatric Research, Shanghai Jiao Tong University School of Medicine, Shanghai, China; ^2^National R&D Branch Center for Freshwater Aquatic Products Processing Technology (Shanghai), Integrated Scientific Research Base on Comprehensive Utilization Technology for By-Products of Aquatic Product Processing, Ministry of Agriculture and Rural Affairs of the People's Republic of China, Shanghai Engineering Research Center of Aquatic-Product Processing and Preservation, College of Food Science & Technology, Shanghai Ocean University, Shanghai, China

**Keywords:** β-carotene, Chinese longsnout catfish (*Leiocassis longirostris Günther*) skin gelatin, emulsion, gel strength, headspace-solid phase microextraction-gas chromatography-mass spectrometry

## Abstract

The exploration of deodorization is important for the application of gelatin in food industry. In this work, the effect of β-cyclodextrin (β-CD) deodorization on the volatile chemicals and functional properties of three types of gelatins (commercial porcine skin gelatin, cold water fish skin gelatin, and Chinese longsnout catfish skin gelatin) were studied. The results suggested the odors of commercial gelatins were significantly less than home-extracted gelatins. The β-CD deodorization efficiency was dependent on both β-CD concentration and volatile chemical. (E)-2-Octenal (C_8_H_14_O), 1-octen-3-ol (C_8_H_16_O), 2-pentyl-furan (C_9_H_14_O), and hentriacontane (C_17_H_36_) could be deodorized at low β-CD concentration (even at 2 mg/mL). The best β-CD deodorization concentration for 66.7 mg/mL of Chinese longsnout catfish skin gelatin was 30 mg/mL. β-CD addition could not change the gel forming ability and emulsion activity of gelatins, whereas it had different and concentration-dependent effects on the emulsion stability of gelatins. β-CD addition had no obvious effects on the droplet sizes, droplet coalescence and liquid-gel transition behaviors, but had different effects on the creaming of the emulsions stabilized by three types of gelatins. The encapsulation of β-carotene did not significantly change the droplet trimodal size distribution and liquid-gel transition of fish oil-loaded emulsions. However, β-carotene might delay the droplet coalescence. The creaming stability of β-carotene/fish oil-loaded gelatin/β-CD-stabilized emulsions was dependent on the gelatins, β-CD, and β-carotene. Finally, the β-carotene retention in the emulsions was dependent not on β-CD addition but on the nature of the gelatins. These results provided useful information to understand the molecular deodorization behaviors and explore the deodorization of emulsifiers for food emulsions.

## Introduction

Gelatins are generally extracted from different animal sources (e.g., mammal, poultry, fishes, and insects) and tissues (e.g., skins, hides, bone, scales, and insect bodies) (1). Due to the unique functional properties, gelatins have been widely explored and applied in the fields of tissue engineering (2), drug delivery (3), cosmetic (4), and food (5). In the field of food, gelatins have been explored and applied as emulsifiers (1), setting agents (6), packaging films (7), etc., Gelatins can be applied as emulsifiers in oil-in-water emulsions, which are integral parts of many foods and beverage (10). In order to improve the emulsifying properties of gelatins, many methods have been applied to modify them such as physical mixing, chemical crosslinking, and enzymatic modification (1, 11, 12). In addition, emulsions are potential dosage systems to encapsulate fish oil for isolating fishy smell (13). The structures and functions of gelatins are dependent on the sources. Therefore, the comparison of gelatins from different sources is necessary for their potential food application.

The extracted gelatins have strong odor of gelatins from the animal sources, which is an important negative factor limiting their food application such as ingredients and supplements (8, 9). Therefore, the deodorization exploration has attracted much and much attention in the field of gelatin science. The development and application of gelatin deodorization methods might improve the stability and reduce the odor of the fish oil-loaded gelatin-stabilized emulsions. Further, it is necessary to explore the effect of the deodorization on the properties of gelatin emulsifiers from different sources.

Cyclodextrins (CDs) are cyclic glucose oligomers with 6–8 glucose units linked by α-1,4-glucosidic bonds. There are α-, β- and γ-CD with 6, 7, and 8, respectively, of glucose units. They can entrap hydrophobic molecules into their hollow centers to form inclusion complexes. β-CD have been widely explored in the fields of tissue engineering (14), drug delivery (15), cosmetic (16), and textile (17). In the field of food science, β-CD has been applied for nutrient encapsulation (18), food 3D printing (19), food packaging (20). The inclusion of curcumin in β-CD drastically increased the water solubility of curcumin (21). The addition of β-CD could mask fishy odor of microencapsulated fish oil (22). Further, β-CD could be applied to lower fishy odor, decrease the gel strength, and increase the emulsion stability index of tiger puffer skin gelatin (23). However, the effects of β-cyclodextrin on different types of gelatins are not illustrated and their effects on the storage and nutrient stability of gelatin-stabilized emulsions are also not investigated until now.

The aim of this work was to analyze the effect of β-cyclodextrin addition on the volatile chemicals and functional properties of three types of gelatins from different sources (commercial porcine skin gelatin, PSG; commercial cold water fish skin gelatin, CFG; and Chinese longsnout catfish (*Leiocassis longirostris Günther*) skin gelatin, CLCSG). PSG, CFG, and CLCSG represent gelatins from mammal, cold water fish, and warm water fish skins, respectively. First, CLCSG was extracted using an acetic acid-pretreatment method. Second, the effect of β-CD deodorization time on the odors of gelatins was analyzed using a headspace-solid phase microextraction-gas chromatography-mass spectrometry (HS-SPME-GC-MS) system. Third, the effect of β-CD deodorization on the structural and functional properties of gelatins were studied. Fourth, the effect of β-CD deodorization on the preparation and stability of fish oil- or β-carotene/fish oil-loaded emulsions was investigated. Final, the effect of β-CD deodorization on the β-carotene stability in the emulsions were analyzed.

## Materials and methods

### Materials

Chinese longsnout catfish (*Leiocassis longirostris Günther*) skin was purchased from Honghu Wannong Aquatic Product and Food Co., Ltd. (Honghu City, Hubei Province, China) and stored at−18°C. PSG (type A, ~ 300 g Bloom) and CFG were purchased from Sigma Aldrich (Shanghai, China). β-CD was purchased from Shanghai Macklin Biochemical Technology Co., Ltd. (Shanghai, China). β-Carotene (purity ≥ 96%) was purchased from Shanghai Aladdin Bio-Chem Technology Co., Ltd. (Shanghai, China). Deep sea fish oil (food grade, DHA + EPA ≥ 70%) was purchased from Xi'an LvTeng Biological Technology Co., Ltd, Shaanxi Province, China. All the other common chemicals were bought from Sinopharm Chemical Reagent Co., Ltd. (Shanghai, China).

### CLCSG extraction

The extraction of CLCSG was performed by an acetic acid-pretreatment method, as described in our previous paper (24). Briefly, frozen fish skins were thawed, cleaned, and cut into skin pieces (0.5 × 0.5 cm). The skin pieces (5 g) were immersed in 0.1 mol/L of NaOH aqueous solution at a skin/solution ratio of 1:10 (g/mL). After 1 h, the skin pieces were rinsed with ultrapure water and soaked in 50 mL of 0.05 mol/L of acetic acid aqueous solution for 3 h. The skin pieces were thoroughly rinsed with ultrapure water and put in 50 mL of ultrapure water. The mixture was vibrated (120 rpm) at 55°C for 6 h to extract CLCSG. Then, the mixture was filtered with a double layer of gauze. The obtained solutions were frozen and freeze-dried to obtain dried CLCSG.

### Gelatin deodorization process

Three types of gelatins (PSG, CFG, and CLCSG) were added in ultrapure water. After 30 min, the mixtures were incubated at 45°C with the shaking speed of 120 rpm for 30 min to obtain dissolved gelatins at a concentration of 66.7 mg/mL. β-CD was added in the solutions with different concentrations. The concentrations were indicated in the results and discussion section. The mixtures were then magnetically stirred (450 rpm) at 50°C for 1 h.

### Headspace-solid phase microextraction-gas chromatography-mass spectrometry

A HS-SPME-GC-MS system was applied to analyze the volatile chemicals after the β-CD deodorization process, as described in section 2.3 (25). A static HS-SPME method was applied to extract volatile compounds of deodorized gelatin samples. Briefly, 10 mL of sample was added in a headspace bottle. The bottle was sealed and immediately equilibrated at 50°C for 15 min. A 50/30 μm DVB/CAR/PDMS SPME fiber (Supelco Company, Bellefonte, PA, United States) was inserted into the headspace bottle to extract the volatile compounds at 50°C for 45 min. Subsequently, the fiber was immediately injected into a GC system (7,890 b, Agilent Technologies, Palo Alto, CA, United States) coupled with a MS system (5,977, Agilent). The fiber was desorbed in the GC injection port for 5 min at 250°C.

The volatile compounds were examined by the MS. A HP-5MS elastic capillary column (30 m length × 0.25 mm internal diameter × 0.25 μm film thickness, Agilent Technologies, Foster City, California, United States) was used. The column oven temperature was programmed as follows: The initial temperature of 40°C (1 min hold) was ramped to 180°C at a speed of 3°C/min, then ramped to 250°C (1 min hold) at a speed of 10°C/min. High-purity helium was used as the carrier gas with a flow rate of 1 ml/min. The ionization voltage was 70 eV. The ion source temperature was 230°C. The transfer line temperature was 230°C. The quadrupole temperature was 150°C. The MS scan was set to m/z 45–550. The mass spectrum was compared with NIST 17.0 MS database (National Institute of Standards and Technology library, United States). The volatile compounds were identified when they had matching and anti-matching degrees of above 800 (maximum 1,000).

### SDS-PAGE

The molecular weight distributions of gelatins with β-CD were studied using sodium dodecyl sulfate-polyacrylamide gel electrophoresis (SDS-PAGE) technique (26). After the deodorization process (section 2.3), the deodorized gelatin (66.7 mg/mL) solutions with β-CD (5 and 30 mg/mL) were freeze-dried to obtain dried samples. Then, 0.02 g of freeze-dried samples were incubated in 10 mL of ultrapure water at 45°C for 1 h. After adjusting pH to 7.0, the solution was mixed with 2× sample loading buffer (Sangon Biotech Co., Ltd., Shanghai, China) at a volume ratio of 1:1 and boiled for 5 min. Then, 10 μL of the sample was loaded into SurePAGE Bis-Tris gel (8% specification, GenScript, Nanjing City, Jiangsu Province, China). Multicolor protein ladder standard (5–270 kDa, Spectra^TM^, GenScript) was used as control. The gel was mounted in a DYCZ-24KS electrophoresis cell (Beijing Liuyi Biotechnology Co., Ltd., Beijing, China) and treated by a voltage of 120 V using a DYY-6D electrophoresis power supply (Beijing Liuyi Biotechnology Co., Ltd.) for 80 min. The gel was stained by a mixture of 0.1% (v/v) Coomassie Brilliant Blue R-250, 25% (v/v) isopropanol, and 10% (v/v) acetic acid for 3 h. After this step, the gel was destained by a mixture of 20% (v/v) ethanol and 10% (v/v) acetic acid. The gel with clear bands was photographed by a digital camera.

### ATR-FTIR spectrometry

The characterized spectra of gelatins with β-CD were studied using attenuated total reflectance Fourier transform infrared (ATR-FTIR) spectrometry (26). After the deodorization process (section 2.3), the deodorized gelatin solutions (66.7 mg/mL) with β-CD (30 mg/mL) were freeze-dried to obtain dried samples. The freeze-dried samples were examined using an ATR-FTIR spectrometer (Spotlight 400, PerkinElmer, Waltham, MA, United States) with a range of 600–4,000 cm^−1^, an average scan of 32, and a resolution of 1 cm^−1^.

### Gel strength

The gel strength values of gelatins was measured using a Model TA-XT Plus Texture Analyzer (Stable Micro Systems Ltd., Surrey, United Kingdom) (26). After the deodorization process (section 2.3), the deodorized gelatin solutions (66.7 mg/mL) with β-CD (5 and 30 mg/mL) were freeze-dried to obtain dried samples. Then, 1.0 g of freeze-dried sample was dissolved into 15 mL of ultrapure in a 25 mL glass breaker water at 45°C for 1 h and incubated at 10°C for 16 h. Then, the gel strength values were determined with a load cell of 50 Kg and a 12.7 mm diameter flat-faced cylindrical Teflon plunger. The maximum force (g) to press the sample to a 4 mm depression at a rate of 1 mm/s was recorded as the gel strength value.

### Emulsion preparation and observation

The fish oil- or β-carotene/fish oil-loaded emulsions stabilized by three types of gelatins with β-CD were prepared using a simple homogenization method (27). After the deodorization process (section 2.3), the deodorized gelatin solutions (66.7 mg/mL) with β-CD (5 and 30 mg/mL) were freeze-dried to obtain dried samples. Then, the freeze-dried samples were dissolved in ultrapure water (10 mg/mL) at 45°C for 1 h and then the pH was adjusted to 9.0. β-Carotene was added into fish oil (0.5 mg/mL of β-Carotene) and was magnetically stirred (300 rpm) overnight to make sure that no obvious precipitates were observed by eyes. Then, the fish oil was centrifuged at a speed of 10,614 × g for 10 min to remove undissolved β-carotene. The oil phase (fish oil or β-Carotene/fish oil) and water phase (gelatin with β-CD) were mixed at volume ratio of 1:2 and then homogenized using a high-speed homogenizer (T10 ULTRA TURRAX homogenizer, 10 mm head, IKA, Guangzhou City, Guangdong Province, China) at a speed of 11,500 rpm for 1 min. The obtained emulsions were stored at 4°C for the storage stability research (β-carotene/fish oil-loaded emulsions were stored in dark). One drop or small piece of the sample was dropped on the glass slide and then was observed by an ML 8,000 upright optical microscope (Shanghai Minz, China). Three images from different batch were analyzed by Image J software 1.53c (Wayne Rasband, National Institutes of Health, United States) to obtain the droplet sizes (500–5,000 droplets). The droplet sizes were treated by Gaussian fitting to get the size distribution. The creaming index (CI) values of these emulsions were obtained by dividing serum layer height by total height and then multiplying by 100.

### Emulsifying parameters

Emulsifying activity index (EAI) and emulsifying stability index (ESI) of three types of gelatins with β-CD were determined using the fish oil-loaded emulsions (28). After the preparation of the emulsions (section 2.8), 20 μL of homogenized emulsions were pipetted at 0 and 10 min, and then diluted with 0.001 g/mL sodium dodecyl sulfate (SDS) solution (1:100, v/v) and Vortexed for 10 s. The ultraviolet-visible spectrophotometer (T6, Beijing Purkinje General Instrument Co., Ltd., Beijing, China) was used to determine the absorbances of the diluted emulsions at 500 nm using SDS solution as the control. The EAI and ESI values were calculated the following formula, respectively:


(1) $$\begin{array}{l} EAI\;\left(m^{2}/g\right)=\frac{2\times 2.303\times A_{0}\times N}{\varphi \times C\times 10000} \end{array}$$

(2) $$\begin{array}{l} ESI\;\left(\min\right)=\frac{A_{0}\times △t}{A_{0}-A_{10}} \end{array}$$

Where *A*_0_ and *A*_10_ were the measured absorbances of the diluted emulsions from the homogenized emulsions at 0 min and 10 min, respectively, *N* is the dilution factor (100), φ is the oil volumetric fraction (0.33), *C* is the weight of protein per unit volume (10 mg/mL), and △*t* is the time interval (10 min).

### β-carotene stability

The β-carotene stability in the emulsions was measured according to a standard curve comparison method. Briefly, 0.0010 g of β-carotene was added into a mixture of 4 mL of methanol and 6 mL of N-hexane. The mixture was Vortexed to allow β-carotene to dissolve into N-hexane phase. After 5 min, the N-hexane phase was extracted and set the volume to 25 mL by adding N-hexane. The obtained β-carotene N-hexane solution (0.04 mg/mL) was diluted by N-hexane to a series of concentrations: 1.5, 2.0, 2.5, 3.0, 3.5, 4.0, 4.5, and 5.0 μg/mL. The absorbances (A, at the range of 0.20–0.80) of these solutions were determined using a UV-Vis spectrometer (T6, Beijing PERSEE General Instrument Co., Ltd., Beijing, China) at 450 nm. The standard curve of UV absorbance (A) as a function of β-carotene concentration (C, μg/mL) was obtained (A = 0.1622 C-0.04, R^2^ = 0.9972).

The β-carotene concentrations of the emulsions during the storage at 4°C in dark was then measured (29). Briefly, 0.5 mL of the β-carotene/fish oil-loaded emulsions was added in a mixture of 4 mL methanol and 6 mL N-hexane. The mixture was Vortexed to allow β-carotene to dissolve into N-hexane phase. After 5 min, the N-hexane phase was extracted. Then, 6 mL of N-hexane was added, the mixture was Vortexed, and the N-hexane phase was extracted. This N-hexane adding-Vortexing-extracting step was repeated once. The N-hexane phases were collected and set the volume to 25 mL by adding N-hexane. The absorbances (A) of these solutions were determined using a UV-Vis spectrometer at 450 nm and the β-carotene concentrations were calculated according the standard curve. Finally, the β-carotene retention percentage was calculated by dividing the β-carotene concentration at the designated time point by the initial β-carotene concentration and multiplying by 100.

### Statistical analysis

All the experiments were repeated three times. All calculated data was expressed as mean value ± standard deviation (SD). One-way ANOVA/Duncan's test (SPSS Statistics 19 software, IBM Corp., Armonk, NY, United States) was employed for the statistical comparison with a *P* < 0.05 level.

## Results and discussion

### Effect of β-CD deodorization on the volatile chemical changes of gelatins

The effects of deodorization on the odors of gelatins were analyzed using a HS-SPME-GC-MS technique (Table 1). Commercial PSG, commercial CFG, and CLCSG showed 6 (3 aldehydes and 3 others), 3 (one phenol and two others), and 17 (6 aldehydes, two phenols, one furans, 8 others), respectively, of volatile chemicals. Tilapia skin gelatin showed 17 volatile chemicals (11 aldehydes, 4 alcohols, and 3 others) (25). In addition, tiger puffer gelatin showed 28 volatile chemicals (5 aldehydes, 2 alcohols, 5 esters, 4 alkenes, 7 alkanes, and 5 others) (23). Therefore, the odors of commercial gelatins were significantly less than home-extracted gelatins.

**Table 1:** Volatile substances of gelatins (PSG, porcine skin gelatin; CFG, cold-water fish skin gelatin; and CLCSG, Chinese longsnout catfish skin gelatin; 66.7 mg/mL) with β-cyclodextrin (β-CD, 0–50 mg/mL) identified using gas chromatography-mass spectrometry.

**No**.	**Compound**	**Molecular formula**	**Peak area / 10** ^**7**^										
			**PSG** + β**-CD (mg/mL)**		**CFG** + β**-CD (mg/mL)**		**CLCSG** + β**-CD (mg/mL)**						
			**0**	**30**	**0**	**30**	**0**	**2**	**5**	**10**	**20**	**30**	**50**
**Aldehydes**	
**1**	(E)-2-Heptenal	C_7_H_12_O	ND	ND	ND	ND	1.55	1.03	1.12	1.04	0.72	ND	ND
**2**	(E)-2-Octenal	C_8_H_14_O	ND	ND	ND	ND	1.87	ND	ND	ND	ND	ND	ND
**3**	Decanal	C_10_H_20_O	0.59	ND	ND	ND	1.74	1.27	1.19	1.01	ND	ND	0.51
**4**	Heptanal	C_7_H_14_O	ND	ND	ND	ND	0.95	0.76	0.64	0.71	0.63	ND	ND
**5**	Hexanal	C_6_H_12_O	0.88	ND	ND	ND	11.3	9.6	9.37	10.1	12.1	7.01	0.35
**6**	Nonanal	C_9_H_18_O	2.35	ND	ND	ND	8.05	6.17	5.39	5.53	4.1	2.53	2.5
	Subtotal		3.82	0	0	0	25.46	18.83	17.71	18.39	17.55	9.54	3.36
**Phenols**	
**7**	2,4-Di-tert-butylphenol	C_14_H_22_O	ND	ND	3.6	ND	0.73	0.81	0.57	ND	ND	ND	ND
**8**	1-Octen-3-ol	C_8_H_16_O	ND	ND	ND	ND	1.23	ND	ND	ND	ND	ND	ND
	Subtotal		0	0	3.6	0	1.96	0.81	0.57	0	0	0	0
**Furans**	
**9**	2-pentyl-Furan	C_9_H_14_O	ND	ND	ND	ND	1.19	ND	ND	ND	ND	ND	ND
	Subtotal		0	0	0	0	1.19	0	0	0	0	0	0
**Others**	
**10**	2,5-Cyclohexadiene-1,4-dione,2,6-bis(1,1-dimethylethyl)-	C_14_H_20_O_2_	ND	ND	ND	ND	0.56	0.7	0.48	ND	ND	ND	ND
**11**	ButylatedHydroxytoluene	C_17_H_24_O_3_	ND	ND	ND	ND	6.54	6.34	6.78	8.56	16.2	29.9	24.1
**12**	2,2,4-Trimethyl-1,3-pentanedioldiisobutyrate	C_16_H_30_O_4_	0.97	ND	0.28	ND	0.95	ND	0.77	ND	ND	ND	ND
**13**	1,1-dimethyl-Hydrazine	CH_4_N_2_O	ND	ND	ND	ND	0.34	ND	ND	ND	ND	ND	0.63
**14**	Oxime-,methoxy-phenyl-	C_8_H_9_NO_2_	6.32	7.32	13.8	1.55	9.71	18.5	21.6	19	18.3	13.3	ND
**15**	Hentriacontane	C_17_H_36_	ND	ND	ND	ND	0.29	ND	ND	ND	ND	ND	ND
**16**	Pentadecane	C_17_H_36_	ND	ND	ND	ND	0.46	ND	0.86	ND	4.66	2.05	1.21
**17**	Tridecane	C_13_H_28_	ND	ND	ND	ND	0.35	0.9	1.95	1.7	0.2	ND	1.35
**18**	Nonadecane	C_19_H_40_	0.31	ND	ND	ND	ND	ND	ND	ND	ND	ND	ND
	Subtotal		7.6	7.32	14.08	1.55	19.2	26.44	32.44	29.26	39.36	45.25	27.29
	Total		11.42	7.32	17.68	1.55	47.81	46.08	50.72	47.65	56.91	54.79	30.65

ND means not detected.

For the gelatins with 30 mg/mL of β-CD addition, one (Oxime-,methoxy-phenyl-), one (Oxime-,methoxy-phenyl-), and 5 volatile chemicals (two aldehydes and 3 others) were detected from the PSG, CFG, and CLCSG, respectively. They suggested β-CD addition could decrease the odors of these three gelatins, which were agreement with the effects of β-CD addition on tilapia skin gelatin (25) and tiger puffer gelatin (23). Therefore, β-CD was an efficient deodorization agent for gelatins.

In order to study the effect of β-CD concentration on the deodorization of different volatile chemicals of CLCSG, we analyzed the volatile chemicals of CLCSG with 0–50 mg/mL of β-CD addition (Table 1). The subtotal areas of each type and the total peak areas of all types were also summarized in Table 1. They did not show obvious metrological relationships to β-CD concentrations. The results suggested the deodorization was dependent on both β-CD concentration and volatile chemical. (E)-2-Octenal (C_8_H_14_O), 1-octen-3-ol (C_8_H_16_O), 2-pentyl-furan (C_9_H_14_O), and hentriacontane (C_17_H_36_) could be deodorized at low β-CD concentration (even at 2 mg/mL), which suggested that these chemicals were easier to be deodorized by β-CD than other volatile chemicals. In addition, decanal (C_10_H_20_O), 1,1-dimethyl-Hydrazine (CH_4_N_2_O), and tridecane (C_13_H_28_) were deodorized at middle β-CD concentrations, whereas were not deodorized at high β-CD concentrations. It suggested an excessive amount of β-CD might decrease the efficiency of deodorization and the best β-CD deodorization concentration for 66.7 mg/mL of CLCSG was 30 mg/mL.

### Effect of β-CD deodorization on the structural and functional characteristics of gelatins

**Figure 1:**
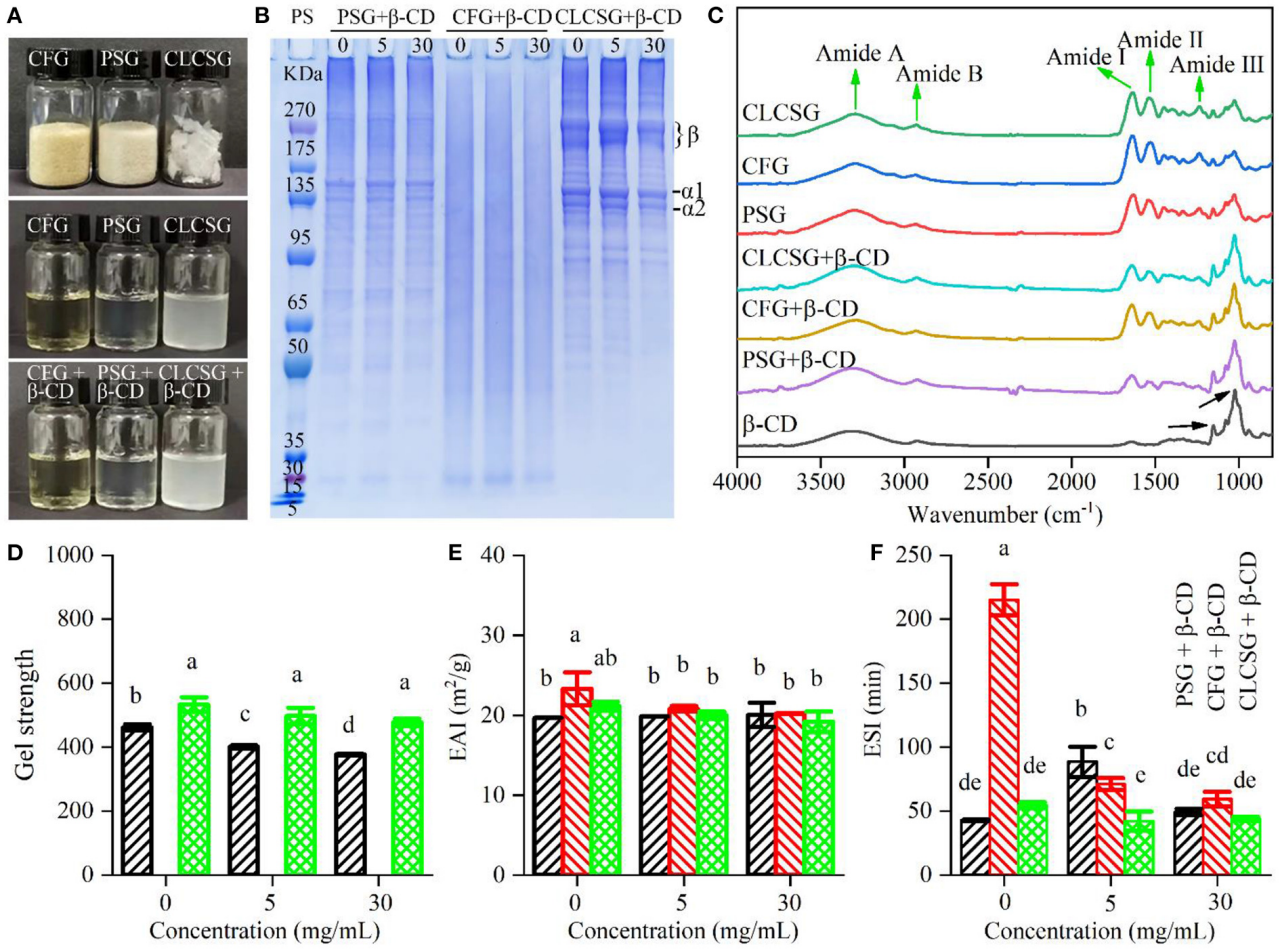
Structural and functional properties of gelatins (PSG, porcine skin gelatin; CFG, cold-water fish skin gelatin; and CLCSG, Chinese longsnout catfish skin gelatin) with β-cyclodextrin (β-CD). **(A)** Digital camera images of solid gelatins, gelatin solutions (66.70 mg/mL), and gelatin solutions (66.70 mg/mL) with β-CD (5 mg/mL). **(B)** SDS-PAGE results. The labels β, α1, and α2 indicates the bands those correspond to β chain, β chain, α1 chain, and α2 chain, respectively, of collagen. **(C)** ATR-FTIR spectra of freeze-dried gelatin with β-CD. **(D–F)** Gel strength values of gelatins with β-CD at different β-CD concentrations (0, 5, and 30, respectively).

The effect of β-CD on the structural characteristics of three types of gelatins were studied. As shown in Figure 1A, the addition of β-CD did not obviously change the appearance of gelatin solutions. SDS-PAGE results (Figure 1B) showed the molecular weight distribution of three types of gelatins were consistent with our previous works (24, 28, 30). Moreover, the addition of β-CD did not obviously change the molecular weight distribution of three types of gelatins, which suggested that no chemical bonds were formed between gelatins and β-CD. The ATR-FTIR spectra of β-CD, gelatins, and gelatins with β-CD were shown in Figure 1C. Three types of gelatins showed similar spectra with five characteristic bands of gelatins (Amide A, Amide B, Amide I, Amide II, and Amide III, indicated by black arrows). Especially, gelatins had three characteristic peaks (Amide I, II, and III bands) that β-CD did not obviously show. β-CD showed two characteristic peaks (1,024 and 1,153 cm^−1^, indicated by black arrows) that gelatins did not more obviously show. The ATR-FTIR spectra of gelatins with β-CD showed both the three characteristic peaks of gelatins and the two characteristic peaks of β-CD, which confirmed the presence of both gelatins and β-CD in the samples. These results (appearances, SDS-PAGE patterns, and ATR-FTIR spectra) confirmed the β-CD deodorization was a result of physical mixing.

The effect of β-CD on the functional characteristics of three types of gelatins were studied. After β-CD addition, both PSG and CLCSG still could form gel, whereas CFG still could not form gel (data not shown). Therefore, β-CD addition could not change the gel forming ability of gelatins. With the increase of β-CD concentration (Figure 1D), PSG/β-CD gels showed decreased gel strength, whereas CLCSG/β-CD gels showed slightly decrease trends (not significant). They were consistent with that β-CD addition could decrease the gel strength of tiger puffer skin gelatin (23). The addition of β-CD might weaken the formation of gelatin network in gels, and therefore, might decrease the gel strength. The β-CD addition had no obvious effect on the EAI values (Figure 1E), which was consistent with the effect of β-CD addition on the EAI value of tiger puffer skin gelatin (23). However, the β-CD addition had different and concentration-dependent effects on the ESI values of three types of gelatins (Fig. 1F), which was different to the effect of β-CD addition on the ESI value of tiger puffer skin gelatin (23).

### Effect of β-CD deodorization on the stability of fish oil-loaded emulsions stabilized by gelatins

**Figure 2:**
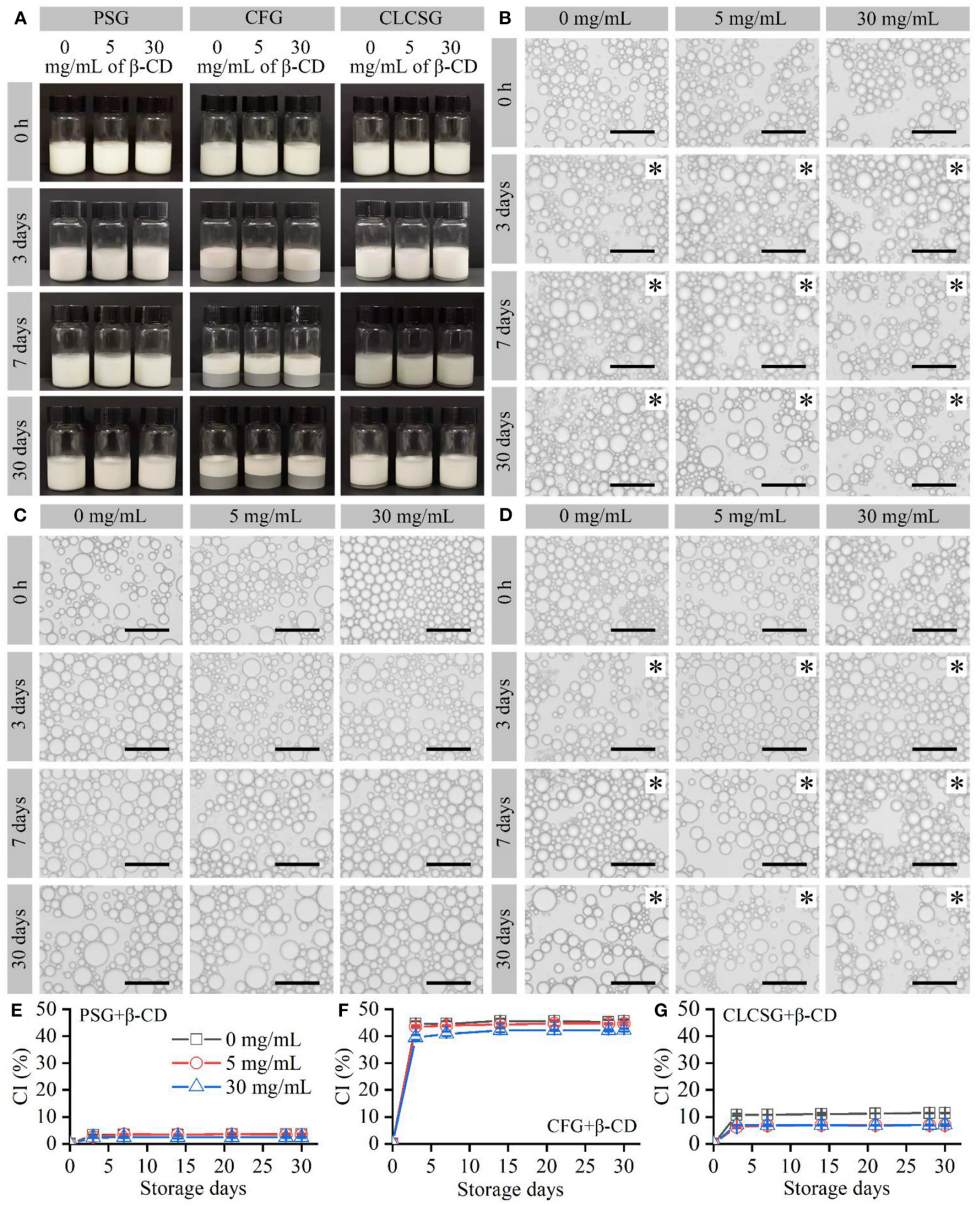
Preparation and storage (4°C) of fish oil-loaded emulsions stabilized by gelatins with β-CD. **(A)** Digital camera images of the emulsions. **(B–D)** Optical microscopy images of the emulsion droplets stabilized by PSG, CFG, and CLCSG, respectively, with β-CD. Black asterisks indicate emulsion gels. Scale bars indicate 50 μm. **(E–G)** Creaming index (CI) values of the emulsions.

Fish oil-loaded emulsions stabilized by three types of gelatins with β-CD were prepared and stored at 4°C. All these freshly-prepared emulsions were milk-white liquids (Figure 2A: 0 h) and consisted of micrometer-sized droplets (Figures 2B–D: 0 h) with trimodal sizes (Figures S1, S2). They were consistent with the previously reported fish oil-loaded gelatin-stabilized emulsions (24, 28, 30). The droplet size distribution analyses (Figures S1, S2) showed the β-CD deodorization of gelatins had no obvious effects on the droplet sizes of the fish oil-loaded emulsions stabilized by three types of gelatins with β-CD.

With the increase of storage time, the sizes of the emulsion droplets slightly increased due to droplet coalescence (27). The liquid emulsions stabilized by PSG, PSG/β-CD, CLCSG, and CLCSG/β-CD changed into emulsion gels at day 3. However, the liquid emulsions stabilized by CFG and CFG/β-CD did not change into emulsion gels even at day 30, which was consistent with previous work on the emulsion stabilized by CFG (28). These results suggested β-CD addition did not obviously change the droplet coalescence and liquid-gel transition behaviors during the storage.

The emulsions showed creaming at day 3 (Figures 2A,E–G). β-CD addition did not obviously change the creaming of the emulsions stabilized by PSG (Figure 2E) and CFG (Figure 2F), whereas it slightly decreased the creaming of the emulsions stabilized by CLCSG (Figure 2G). The creaming index (CI) values of the emulsions were mainly dependent on the gelatins: PSG ≈ PSG/β-CD < CLCSG ≈ CLCSG/β-CD < CFG ≈ CFG/β-CD. This trend was consistent with previous work that CFG induced significantly higher CI values than bovine bone gelatin for fish oil-loaded emulsions (28).

### Effect of β-CD deodorization on the stability of β-carotene/fish oil-loaded emulsions stabilized by gelatins

**Figure 3:**
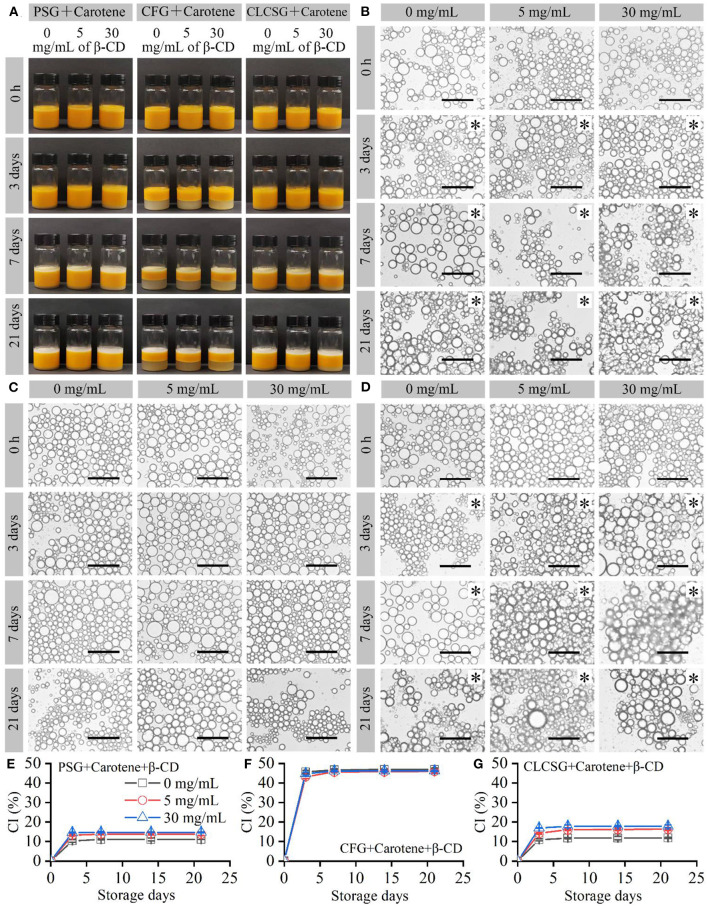
Preparation and storage (4°C) of β-carotene/fish oil-loaded emulsions stabilized by gelatins with β-CD. **(A)** Digital camera images of the emulsions. **(B–D)** Optical microscopy images of the emulsion droplets stabilized by PSG, CFG, and CLCSG, respectively, with and β-CD. Black asterisks indicate emulsion gels. Scale bars indicate 50 μm. **(E–G)** CI values of the emulsions.

β-Carotene/fish oil-loaded emulsions stabilized by three types of gelatins with β-CD were prepared and stored at 4°C. All these freshly-prepared emulsions were yellow liquids (Figure 3A: 0 h) and consisted of micrometer-sized droplets (Figures 3B–D: 0 h) with trimodal sizes (Figures S3, S4). The color was consistent with previously reported β-Carotene/soybean oil-loaded emulsion (31). Therefore, the yellow color was resulted from the presence of β-carotene. The trimodal droplet size distribution was consistent with the previously reported fish oil-loaded gelatin-stabilized emulsions (24, 28, 30) and fish oil-loaded gelatin/β-CD-stabilized emulsions (Figure 2). The droplet size distribution analyses (Figures S3, S4) showed the β-CD deodorization of gelatins had no obvious effects on the droplet sizes of the β-carotene/fish oil-loaded emulsions stabilized by three types of gelatins with β-CD. In addition, the β-carotene/fish oil-loaded emulsions (Figure S4) did not show significant droplet size differences to the fish oil-loaded emulsions (Figure S2).

With the increase of storage time, the sizes of the β-carotene/fish oil-loaded emulsion droplets did not show obvious increase (Figures 3B–D). It was different to the fish oil-loaded emulsion droplet size, which increased due to droplet coalescence (Figures 2B–D) (27). Therefore, the presence of β-carotene might delay the droplet coalescence. The β-carotene/fish oil-loaded liquid emulsions stabilized by PSG, PSG/β-CD, CLCSG, and CLCSG/β-CD changed into emulsion gels at day 3. However, the β-carotene/fish oil-loaded liquid emulsions stabilized by CFG and CFG/β-CD did not change into emulsion gels even at day 30. Compared with the fish oil-loaded gelatin/β-CD-stabilized emulsions (Figure 2), the presence of β-carotene did not obvious affect the emulsion liquid-gel transition.

The β-carotene/fish oil-loaded emulsions showed creaming at day 3 (Figures 3A,E–G), which were similar to fish oil-loaded emulsions (Figure 2). The presence of β-carotene increased the CI values of PSG- and PSG/β-CD-stabilized emulsions (Figure 3E vs. Figure 2E) and did not obviously affect the CI values of CFG- and CFG/β-CD-stabilized emulsions (Figure 3F vs. Figure 2F). Moreover, the presence of β-carotene increased the CI values of CLCSG/β-CD-stabilized emulsions and did not obviously affect the CI values of CLCSG-stabilized emulsions (Figure 3G vs. Figure 2G). The creaming index (CI) values of the β-carotene/fish oil-loaded emulsions were dependent on the gelatins and β-CD: PSG ≈ CLCSG < PSG/β-CD < CLCSG/β-CD < CFG ≈ CFG/β-CD). This order was different to the CI values of the fish oil-loaded gelatin/β-CD-stabilized emulsions (Figures 2E–G). Therefore, the creaming stability was dependent on the gelatins, β-CD, and β-carotene.

### Effect of β-CD deodorization on the retention of β-carotene in the fish oil-loaded emulsions stabilized by gelatins

**Figure 4:**
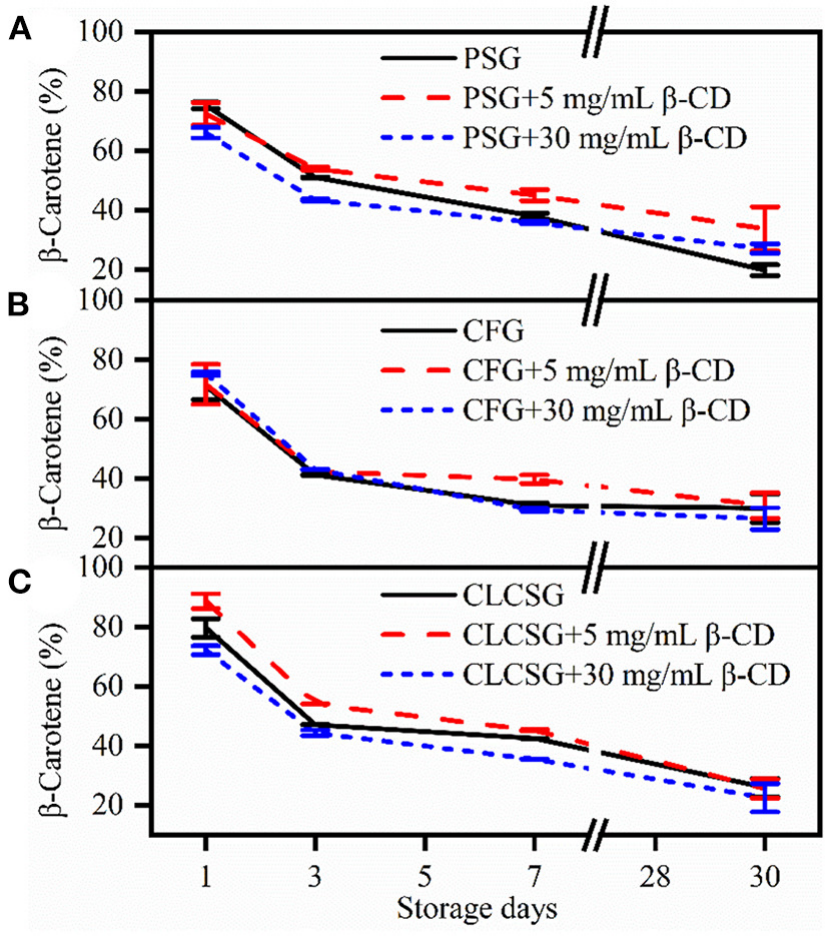
Stability of β-carotene in fish oil-loaded emulsions stabilized by gelatins (**A**: PSG; **B**: CFG; **C**: CLCSG) with β-CD at 4°C.

The β-carotene retention percentages of the β-carotene/fish oil-loaded emulsions stabilized by gelatin/β-CD were analyzed during the storage at 4°C (Figure 4). The β-carotene retention percentages decreased with the increase of storage time. This β-carotene retention percentage trend was consistent with that of casein-stabilized emulsions (32) and wheat gluten nanoparticle-xanthan gum-stabilized Pickering emulsions (33). There was no obvious metrological relationship between β-CD concentrations and β-carotene retention percentages. After 30 days storage, PSG (19.8 ± 1.8%, Figure 4A) induced lower β-carotene retention percentages than aquatic gelatins (CFG: 29.9 ± 4.8%, Figure 4B CLCSG: 26.0 ± 3.1%, Figure 4C). The presence of β-CD (both 5 and 30 mg/mL) could increase the β-carotene retention percentages (33.7 ± 7.4% and 27.1 ± 1.6%) in the emulsion stabilized by PSG after 30 days storages (Figure 4A). However, the presence of β-CD (both 5 and 30 mg/mL) did not obviously affect the β-carotene retention in the emulsions stabilized by aquatic gelatins (CFG: Figure 4B; and CLCSG: Figure 4C). Therefore, the β-carotene retention was dependent not on β-CD addition but on the nature of the gelatins.

The β-carotene in casein-stabilized emulsions showed a 100% degradation after 30 days storage at 50°C (32). The β-carotene in wheat gluten nanoparticle-xanthan gum-stabilized Pickering emulsions showed 94.2 and 70.1% retention percentages after 30 days storage at 25°C and 37°C, respectively. Moreover, it showed 38.5% retention percentage after 14 days storage at 60°C (33). It suggested the β-carotene retention percentages decreased with the increase of storage temperature. According to our and these previous works, the β-carotene retention was dependent on the emulsifiers of the emulsions. Moreover, gelatins induced less β-carotene retention ability than casein and wheat gluten nanoparticle-xanthan gum.

## Conclusion

In this work, the effect of β-CD deodorization on the volatile chemicals and functional properties of three types of gelatins were studied. All these results suggested that β-CD was an efficient deodorization agent for gelatins. Moreover, the deodorized gelatins with β-CD can be used to stabilize fish oil- or β-carotene/fish oil-loaded emulsions. The β-CD addition did not change the β-carotene retention in the emulsions. These results provided useful information to understand the molecular deodorization behaviors of gelatins. It also provided useful information to guide the deodorization of emulsifiers for food emulsions. However, the molecular interaction mechanism between β-CD and different volatile chemicals were not analyzed to illustrate the molecular deodorization mechanism. Further works are required to analyze more functional (e.g., rheological behaviors) changes and illustrate molecular interaction mechanism, which will be helpful to guide development and application of β-CD-based deodorization agents in the future.

## Data availability statement

The raw data supporting the conclusions of this article will be made available by the authors, without undue reservation.

## Author contributions

LY: investigation, data curation, and writing—original draft. YZ, CS, JC, and JX: investigation. XW: resources. JZ: conceptualization, supervision, data curation, and writing—review editing. All authors contributed to the article and approved the submitted version.

## Funding

This research has been supported by a research grant from the National Key Research & Development Program of China (No. 2019YFD0902003).

## Conflict of interest

The authors declare that the research was conducted in the absence of any commercial or financial relationships that could be construed as a potential conflict of interest.

## Publisher's note

All claims expressed in this article are solely those of the authors and do not necessarily represent those of their affiliated organizations, or those of the publisher, the editors and the reviewers. Any product that may be evaluated in this article, or claim that may be made by its manufacturer, is not guaranteed or endorsed by the publisher.
