# Supplementary material for: Effect of β-cyclodextrin deodorization on the volatile chemicals and functional properties of three types of gelatins
Source: Front Nutr. 2022 Nov 7;9:1059403. doi: 10.3389/fnut.2022.1059403 (PMC9676480; doi:10.3389/fnut.2022.1059403)
Supplement: Supplementary file 1 [file Data_Sheet_1.docx]

**Supplementary Material**

**Effect of β-cyclodextrin deodorization on the volatile chemicals and functional properties of three types of gelatins**

Lili Yang^1,2^, Ye Zi^1,2^, Cuiping Shi^1^, Jiahui Chen^1,2^, Jiamin Xu^1,2^, Xichang Wang^2^, Jian Zhong^1,2,*^

^1^Xinhua Hospital, Shanghai Institute for Pediatric Research, Shanghai Key Laboratory of Pediatric Gastroenterology and Nutrition, Shanghai Jiao Tong University School of Medicine, Shanghai 200092, China

^2^National R&D Branch Center for Freshwater Aquatic Products Processing Technology (Shanghai), Integrated Scientific Research Base on Comprehensive Utilization Technology for By-Products of Aquatic Product Processing, Ministry of Agriculture and Rural Affairs of the People's Republic of China, Shanghai Engineering Research Center of Aquatic-Product Processing and Preservation, College of Food Science & Technology, Shanghai Ocean University, Shanghai 201306, China

^*^Corresponding author at: Xinhua Hospital, Shanghai Institute for Pediatric Research, Shanghai Key Laboratory of Pediatric Gastroenterology and Nutrition, Shanghai Jiao Tong University School of Medicine, Shanghai 200092, China

E-mail: jzhong@shsmu.edu.cn (J. Zhong)

Abbreviated running title: β-cyclodextrin deodorization for gelatin emulsions





**Fig. S1.** Droplet size distribution of freshly-prepared fish oil-loaded emulsions stabilized by gelatins with β- cyclodextrin (β-CD).





**Fig. S2.** The most representative droplet size distribution of freshly-prepared fish oil-loaded emulsions stabilized by gelatins with β- cyclodextrin (β-CD).





**Fig. S3.** Droplet size distribution of freshly-prepared β-carotene/fish oil-loaded emulsions stabilized by gelatins with β-CD.





**Fig. S4.** The most representative droplet size distribution of freshly-prepared β-carotene/fish oil-loaded emulsions stabilized by gelatins with β-CD.
